# Supplementary material for: Thermal Death Kinetics of Three Representative Salmonella enterica Strains in Toasted Oats Cereal
Source: Microorganisms. 2022 Aug 4;10(8):1570. doi: 10.3390/microorganisms10081570 (PMC9416204; doi:10.3390/microorganisms10081570)
Supplement: Supplementary file 1 [file microorganisms-10-01570-s001.zip › microorganisms-1827080-supplementary.pdf]

Supplementary Material

Thermal Death Kinetics of Three Representative *Salmonella enterica* Strains in Toasted Oat Cereal

Matthew Chick [1], Antonio Lourenco [2], Alice Maserati [1], Ryan C. Fink [3,4] and Francisco Diez-Gonzalez [5,*]

Table S1. Thermal inactivation rates of *Salmonella enterica* serovar Typhimurium in toasted oats cereal (TOC) affected by water activity (a_w) and temperature. Decimal reduction time (D -values) calculated by the Linear model and first decimal reduction time (δ -value) and re-estimated first decimal reduction time (Mod. δ -value) for the fixed β, calculated with the Weibull model.

a_w	Temp (◦C)	Linear model parameters			Weibull model parameters								
		D (min)	D SE	R^2_{adj}	δ (min)	δ SE	β	β SE	R^2_{adj}	$\overline{\beta}$ #	Mod. δ (min)	Mod. δ SE	R^2_{adj}
**0.11**	85	150.6	10.33	0.76	65.1	18.09	0.56	0.08	0.81	0.44	40.95	15.06	0.80
	95	28.3*	2.23	0.72	14.4*	5.22	0.59	0.10	0.79		4.82	2.01	0.83
	100	15.9*	2.05	0.63	0.9	0.70	0.32	0.06	0.87		2.70	1.49	0.85
	105	5.9*	0.77	0.63	0.40*	0.27	0.36	0.06	0.87		0.81	0.44	0.86
**0.33**	70	170.9*	12.19	0.75	102.16*	30.90	0.70	0.12	0.77	0.40	31.52	18.22	0.72
	75	88.47*	5.87	0.77	21.36	7.19	0.48	0.06	0.86		12.71	5.39	0.85
	80	26.7*	2.35	0.62	2.09	1.39	0.35	0.06	0.77		3.36	1.86	0.77
	85	5.8*	0.46	0.81	0.55	0.18	0.38	0.03	0.97		0.65	0.20	0.97
**0.53**	65	192.3	6.95	0.96	186.10	19.04	0.97	0.09	0.96	0.64	111.30	23.13	0.93
	70	87.8	6.18	0.82	46.44	12.84	0.64	0.10	0.86		12.87	12.87	0.86
	75	35.4	3.87	0.63	5.41	3.56	0.35	0.08	0.79		18.14	6.73	0.73
	80	7.7	0.81	0.66	3.62	1.70	0.61	0.14	0.69		3.93	1.77	0.69

* $f > F_{table}$

\# $\overline{\beta}$ was calculated using β values for conditions where $f < F_{table}$

Table S2. Thermal inactivation rates of *Salmonella enterica* serovar Tennessee in toasted oats cereal (TOC affected by water activity (a_w) and temperature. Decimal reduction time (D -values) calculated by the Linear model and first decimal reduction time (δ -value) and re-estimated first decimal reduction time (Mod. δ -value) for the fixed β, calculated with the Weibull model.

a_w	Temp (°C)	Linear model parameters			Weibull model parameters								
		D (min)	D SE	R^2_{adj}	δ (min)	δ SE	β	β SE	R^2_{adj}	$\overline{\beta}$ #	Mod. δ (min)	Mod. δ SE	R^2_{adj}
**0.11**	85	148.57	11.18	0.77	105.10	26.05	0.73	0.13	0.78	0.55	70.90	23.97	0.77
	95	24.38	2.02	0.72	5.92	2.35	0.49	0.07	0.82		7.72	2.76	0.81
	100	12.78*	0.97	0.73	5.55*	2.07	0.62	0.10	0.76		4.22	1.79	0.76
	105	4.65	0.57	0.51	0.82	0.50	0.43	0.09	0.68		1.48	0.70	0.67
**0.33**	70	194.86	17.16	0.82	118.08	32.66	0.69	0.12	0.88	0.41	43.77	19.66	0.89
	75	93.48	12.69	0.58	16.45	15.01	0.43	0.13	0.67		14.53	13.93	0.67
	80	30.73	4.37	0.47	2.32	3.26	0.27	0.10	0.68		3.76	2.85	0.70
	85	22.34	5.49	0.36	1.01	2.36	0.26	0.15	0.72		1.99	2.31	0.71
**0.53**	65	224.87	47.45	0.39	112.02	113.13	0.56	0.33	0.39	0.53	103.27	110.18	0.39
	70	195.98	34.53	0.44	66.13	68.18	0.49	0.22	0.45		76.36	73.34	0.45
	75	41.52	3.03	0.71	12.33	3.53	0.55	0.06	0.82		11.36	3.38	0.82
	80	8.59*	1.49	0.40	3.20*	3.30	0.48	0.24	0.42		3.30	2.99	0.42

* $f > F_{table}$

$\overline{\beta}$ was calculated using β values for conditions where $f < F_{table}$

Table S3. Thermal inactivation rates of *Salmonella enterica* serovar Agona in toasted oats cereal (TOC) affected by water activity (a_w) and temperature. Decimal reduction time (D -values) calculated by the Linear model and first decimal reduction time (δ -value) and re-estimated first decimal reduction time (Mod. δ -value) for the fixed β, calculated with the Weibull model.

a_w	Temp (°C)	Linear model parameters			Weibull model parameters								
		D (min)	D SE	R^2_{adj}	δ (min)	δ SE	β	β SE	R^2_{adj}	$\overline{\beta}$ #	Mod. δ (min)	Mod. δ SE	R^2_{adj}
**0.11**	85	201.53	28.28	0.47	63.87	43.35	0.41	0.13	0.54	0.44	71.14	45.23	0.54
	95	64.97	11.59	0.33	41.39	21.97	0.50	0.22	0.36		37.82	22.57	0.36
	100	22.07	2.01	0.70	5.44	2.29	0.42	0.07	0.81		5.90	2.39	0.81
	105	5.33	0.51	0.69	3.62*	1.36	0.77	0.16	0.69		1.16	0.84	0.64
**0.33**	70	197.55	18.42	0.67	42.19	24.08	0.43	0.09	0.75	0.50	60.97	29.76	0.75
	75	87.18	7.34	0.70	29.27	12.43	0.46	0.09	0.80		26.44	9.70	0.81
	80	27.64	2.55	0.72	9.23*	4.55	0.53	0.11	0.80		6.95	3.54	0.81
	85	6.32	0.42	0.85	2.87	0.93	0.61	0.09	0.90		1.49	0.58	0.90
**0.53**	65	144.14*	13.01	0.64	97.78*	32.92	0.74	0.16	0.65	0.61†	71.81		0.64
	70	70.66*	5.64	0.84	49.94*	13.83	0.75	0.14	0.85		37.03		0.84
	75	29.89*	1.26	0.93	17.52*	2.50	0.70	0.05	0.95		13.76		0.95
	80	8.59	1.69	0.33	0.23	0.48	0.25	0.12	0.54		3.52		0.45

* $f > F_{table}$

$\overline{\beta}$ was calculated using β values for conditions where $f < F_{table}$

† value calculated as average of all β values obtained for a_w 0.53

Table S4. Thermal inactivation rates of *Salmonella enterica* serovar Typhimurium in toasted oats cereal (TOC) affected by water activity (a_w) and temperature in the presence of 25% sucrose. Decimal reduction time (D -values) calculated by the Linear model and first decimal reduction time (δ -value) and re-estimated first decimal reduction time (Mod. δ -value) for the fixed β, calculated with the Weibull model.

a_w	Temp (◦C)	Linear model parameters			Weibull model parameters								
		D (min)	D SE	R^2_{adj}	δ (min)	δ SE	β	β SE	R^2_{adj}	$\bar{\beta}$ #	Mod. δ (min)	Mod. δ SE	R^2_{adj}
**0.11**	85	49.50	3.26	0.84	21.60	4.85	0.54	0.06	0.91	0.44	14.98	4.39	0.90
	90	19.25	1.31	0.84	6.52	1.55	0.49	0.05	0.93		5.34	1.45	0.93
	95	6.83	0.74	0.64	0.59	0.31	0.27	0.04	0.90		1.96	0.63	0.86
	100	3.20	0.22	0.84	1.00	0.21	0.46	0.04	0.95		0.92	0.20	0.95
**0.33**	75	84.96	5.85	0.82	21.55	5.80	0.44	0.05	0.93	0.40	17.02	5.21	0.93
	80	45.81	3.87	0.76	11.55	3.91	0.47	0.06	0.90		7.61	3.04	0.90
	85	13.18	1.06	0.76	1.76	0.36	0.36	0.04	0.93		2.44	0.73	0.92
	90	4.28	0.38	0.72	0.39	0.33	0.33	0.03	0.94		NF	NF	NF
**0.53**	70	78.88*	6.12	0.84	35.92	10.81	0.59	0.09	0.90	0.45	19.87	8.31	0.89
	75	35.78	2.58	0.75	10.65	2.70	0.50	0.05	0.92		6.62	1.63	0.94
	80	12.57	1.08	0.81	3.35	1.29	0.43	0.06	0.93		2.61	0.76	0.96
	85	3.88	0.43	0.70	0.14	0.05	0.29	0.02	0.98		0.69	0.22	0.94

* $f > F_{table}$

\# $\bar{\beta}$ was calculated using β values for conditions where $f < F_{table}$

NF = did not fit the average β

Table S5. Thermal inactivation rates of *Salmonella enterica* serovar Tennessee in toasted oats cereal (TOC) affected by water activity (a_w) and temperature in the presence of 25% sucrose. Decimal reduction time (D -values) calculated by the Linear model and first decimal reduction time (δ -value) and re-estimated first decimal reduction time (Mod. δ -value) for the fixed β, calculated with the Weibull model.

**a_w**	**Temp (◦C)**	**Linear model parameters**			**Weibull model parameters**								
		**D (min)**	**D SE**	R^2_{adj}	**δ (min)**	**δ SE**	**β**	**β SE**	R^2_{adj}	$\bar{\beta}$ [#]	**Mod. δ (min)**	**Mod. δ SE**	R^2_{adj}
**0.11**	85	44.93	3.97	0.83	12.04	3.86	0.45	0.06	0.94	0.47	12.98	4.00	0.94
	90	15.61	1.05	0.85	6.61	1.86	0.58	0.08	0.89		4.13	1.54	0.89
	95	6.25	0.65	0.73	1.15	0.53	0.38	0.06	0.88		1.87	0.70	0.87
	100	3.27	0.32	0.77	0.88	0.33	0.45	0.07	0.89		0.97	0.34	0.89
**0.33**	75	83.11	5.35	0.85	39.65*	9.67	0.58	0.07	0.90	0.47	25.30	7.82	0.89
	80	29.32	1.79	0.87	13.27	3.14	0.61	0.07	0.91		7.58	2.58	0.90
	85	12.73	1.09	0.75	4.61	1.78	0.50	0.09	0.85		3.28	1.29	0.86
	90	4.45	0.44	0.60	0.57	0.26	0.30	0.05	0.84		1.51	0.44	0.80
**0.53**	70	83.76	5.28	0.85	42.10*	9.97	0.61	0.08	0.89	0.48	26.60	8.32	0.88
	75	26.52	1.50	0.88	8.85	1.96	0.56	0.05	0.95		6.08	1.66	0.94
	80	9.47	0.67	0.84	2.63	0.76	0.49	0.06	0.93		2.54	0.75	0.93
	85	3.10	0.24	0.78	0.39	0.11	0.38	0.03	0.95		0.74	0.17	0.94

[*] $f > F_{table}$

[#] $\bar{\beta}$ was calculated using β values for conditions where $f < F_{table}$

Table S6. Thermal inactivation rates of *Salmonella enterica* serovar Agona in toasted oats cereal (TOC) affected by water activity (a_w) and temperature in the presence of 25% sucrose. Decimal reduction time (D -values) calculated by the Linear model and first decimal reduction time (δ -value) and re-estimated first decimal reduction time (Mod. δ -value) for the fixed β, calculated with the Weibull model.

a_w	Temp (◦C)	Linear model parameters			Weibull model parameters								
		D (min)	D SE	R^2_{adj}	δ (min)	δ SE	β	β SE	R^2_{adj}	$\overline{\beta}$ #	Mod. δ (min)	Mod. δ SE	R^2_{adj}
**0.11**	85	64.85	4.25	0.84	20.44	3.80	0.44	0.04	0.95	0.41	17.42	3.60	0.95
	90	24.98	1.84	0.81	5.46	1.24	0.39	0.04	0.95		6.10	1.31	0.95
	95	8.16	0.57	0.81	2.34	0.50	0.41	0.04	0.94		2.16	0.45	0.94
	100	4.96	0.39	0.78	1.26	0.36	0.40	0.05	0.91		1.31	0.37	0.91
**0.33**	75	97.26	5.89	0.86	35.89	6.39	0.48	0.04	0.95	0.45	31.86	6.15	0.95
	80	45.51	3.87	0.74	10.81	3.94	0.38	0.06	0.90		13.58	4.05	0.89
	85	13.25	0.78	0.86	4.44	1.04	0.52	0.05	0.93		3.21	0.90	0.93
	90	3.56	0.24	0.82	0.88	0.15	0.43	0.03	0.96		0.98	0.16	0.96
**0.53**	70	81.18*	6.18	0.79	30.70*	10.52	0.53	0.09	0.85	0.48	24.50	9.52	0.85
	75	32.32	2.43	0.85	5.40	1.10	0.42	0.03	0.98		7.77	1.47	0.98
	80	9.62*	0.75	0.84	2.65	1.13	0.54	0.08	0.89		1.94	0.95	0.89
	85	2.74*	0.27	0.75	0.64	0.32	0.47	0.08	0.84		0.68	0.33	0.84

* $f > F_{table}$

$\overline{\beta}$ was calculated using β values for conditions where $f < F_{table}$
